# Effects of Resistance Training on Immunoinflammatory Response, TNF-Alpha Gene Expression, and Body Composition in Elderly Women

**DOI:** 10.1155/2018/1467025

**Published:** 2018-10-28

**Authors:** Luís Ângelo Macêdo Santiago, Lídio Gonçalves Lima Neto, Guilherme Borges Pereira, Richard Diego Leite, Cristiano Texeira Mostarda, Janaina de Oliveira Brito Monzani, Wandson Rodrigues Sousa, Aruanã Joaquim Matheus Rodrigues Pinheiro, Francisco Navarro

**Affiliations:** ^1^Department of Medicine, Federal University of Maranhão (UFMA), Pinheiro, MA, Brazil; ^2^Laboratory of Immunology and Microbiology of Respiratory Infections, LAMIR, Universidade CEUMA, São Luis, MA, Brazil; ^3^Undergraduate Program in Physical Education at the Catholic University of Brasilia, Brasília, Brazil; ^4^Department of Physical Education, Federal University of Espírito Santo (UFES), Vitória, ES, Brazil; ^5^Department of Physical Education, Federal University of Maranhão (UFMA), São Luis, MA, Brazil; ^6^Postgraduate Program in Adult and Child Health, PPGSAC, UFMA, São Luis, MA, Brazil

## Abstract

The aim of this study was to determine the effects of resistance training on the immunologic response, body composition, tumor necrosis factor-alpha (TNF-alpha) gene expression obtained from blood leukocytes, and the cytokines interleukin-6, TNF-alpha, and C-reactive protein (CRP), in the elderly women (mean age 63 ± 2 y). A randomized controlled trial was performed using a bi-set training method for eight weeks in nineteen elderly women. Peripheral blood samples were collected by puncture in pretraining (Pre) and posttraining (Post) moments. In the resistance training group, there was a statistically significant decrease from 38.43 ± 9.48 pg/mL to 11.76 ± 5.19 pg/mL (*p*=0.01) in the serum levels of interleukin-6. Considering serum levels of TNF-alpha, there was a statistically significant difference, comparing the resistance training group at Pre (66.27 ± 10.31 pg/mL) and Post (37.85 ± 9.05 pg/mL) moments (*p*=0.01). In molecular analysis of TNF-alpha gene expression, there was a statistically significant decrease (*p*=0.007) between Pre (0.010 ± 0.01 ng/ml) and Post (0.0002 ± 0.0001 ng/ml) moments. Among CRP data, in the resistance training group, there was a statistically significant reduction, between Pre (2.04 ± 0.32 mg/L) and Post (0.90 ± 0.22 mg/L) moments (*p*=0.001). In the Control group, there was no statistical significance between these two moments. Therefore, the resistance training demonstrated changes in the TNF-alpha gene expression in elderly women, as well as decreased serum levels of interleukin-6, TNF-alpha, and CRP. Such conditions may be related to immune modulation and anti-inflammatory effects, since resistance training releases cytokines, especially interleukin-6, which acts as a TNF-alpha antagonist during exercise.

## 1. Introduction

In recent years, life expectancy has grown considerably. Nowadays, there are 14.3 million Brazilians over 60 years. Until the year 2025, Brazil will become the sixth country in the number of elderly people [1]. Due to this increase in life expectancy and aging, many researchers have investigated several aspects about this theme [2].

Human aging is a process characterized by biological and physiological changes with repercussion on body composition and onset of chronic systemic inflammation, pointed by the increase of inflammatory markers such as interleukin-6 (IL-6), tumor necrosis factor-alpha (TNF-*α*), and C-reactive protein (CRP) [2, 4]. According to Silva and Mura [5], several diseases in this life stage result in changes in the immune system with consequent disturbance in cytokine homeostasis.

Cytokines are proteins that respond to pathogens with cells of innate and adaptive immunity and also act on inflammatory processes [6]. Among cytokines, TNF-*α* is the main mediator of acute inflammatory response whose physiological function is stimulation of leukocytes, signaling to sites of inflammation and activating them in order to eradicate microorganisms and reduce inflammation [3, 7].

Another important cytokine in the inflammatory process is IL-6. It is synthesized from mononuclear phagocytes, vascular endothelial cells, fibroblasts, and adipose and muscle tissues. Its function is stimulation of neutrophil production by bone marrow progenitors, promoting synthesis of proinflammatory cytokines and inhibiting TNF-*α*. Thus, the body returns to its resting state as the inflammatory process is eradicated [6].

C-reactive protein is a risk marker for cardiovascular disease, which also features an important function in the inflammatory process, especially in the acute phase, when CRP can increase up to 1000 times its normal serum level. This protein is produced and released into the bloodstream by hepatocytes, but it can also be produced by arterial walls. Its synthesis is stimulated by IL-1 and IL-6, which are released by macrophages [5]. Among several functions attributed to CRP, perhaps the most important is the synthesis of phagocytic components, which remove structures from blood circulation and induce an anti-inflammatory effect. These events promote tissue repair [8–10].

Several systemic, tissue, cellular, and molecular changes happen in the aging process, featured by progressive dysfunction of the immune system, with an imbalance in the concentrations of pro- and anti-inflammatory cytokines and other inflammatory markers associated with the development of cardiovascular diseases, and the increase in morbidity and mortality, in the elderly [11, 12]. Resistance training is a modality of exercise to prevent diseases, especially cardiovascular diseases and type 2 diabetes mellitus [13]. Thus, some studies suggest that the resistance training is effective in decreasing serum concentrations of proinflammatory cytokines (TNF-*α*, IL-1*β*, and IL-8), being also recommended for health promotion in the elderly [13–16].

Therefore, the role of immunological and inflammatory markers such as TNF-*α*, IL-6, and CRP in the face of chronic resistance training in the elderly can be a good tool for several health professionals and, especially, for the general population, because of the beneficial effects of exercise in this senile age. Thus, this study is mainly aimed at analyzing the immunological response, gene expression, inflammatory markers through cytokines such as TNF-*α*, IL-6, and CRP, and body composition, in a group of elderly women, so as to better understand these events.

Therefore, the role of immunological and inflammatory markers such as TNF-*α*, IL-6, and CRP in relation to resistance training in the elderly can be a good appliance for several health professionals, and especially for the general population, as these effects will be beneficial in this age by reducing proinflammatory cytokines and thereby reducing various types of chronic degenerative diseases due to aging. Thus, this study is aimed at analyzing the behavior of the immune system, through gene expression, inflammatory markers of TNF-*α*, IL-6, and CRP, and body composition in a group of elderly women to better understand these events.

## 2. Materials and Methods

### 2.1. Study Characteristics

It is an experimental and controlled study. The Ethics and Research Committee of the CEUMA University, through the Brazil Platform Ministry of Health, National Health Council, National Commission of Ethics in Research-Division of Experimental Research Involving Human Beings, approved this study under CAEE permit number 10863313.2.1001.5084 (protocol no. 372453/2013). All participants signed an individual consent form, so their identification is included in this article. All procedures performed in this study were in accordance with the Ethical Standards of the Institutional and/or National Research Committee and with the Helsinki Declaration of 1975 and its later amendments or comparable ethical standards.

### 2.2. Sample Selection

This is a nonprobabilistic sample. A list with names and telephone contacts of women aged between 60 and 70 was initially requested from the Integrated Seniors University (UNITI/UFMA). From the telephone contacts available, subjects were invited to take part in the study, and those who had not entered any other structured RT program in the last six months were selected. A meeting was held for explanation about project logistics, for delivering the schedule of activities to all participants, and for the consent form to be signed by them. Also, there were scheduled days, times, and locations for anthropometric evaluation and blood collection. For inclusion criteria, participants should be between 60 and 70 years; not be enrolled in structured RT programs in the last six months; be nonsmokers; have no degree of obesity according to the World Health Organization [17], using body mass index (BMI) and waist-to-hip ratio (WHR) as parameters (BMI ≤ 30 kg/m^2^ and WHR ≤ 1.00); and voluntarily sign the consent term. For exclusion criteria, participants should not have untreated hypertension or diabetes mellitus (these criteria were controlled through directed anamnesis and measurement of changes in total leukocyte numbers, which could indicate acute infectious processes). The total initial sample consisted of 23 elderly women. After eight weeks of RT, the final sample was 19 participants. Two people from the initial sample presented leukocyte values above the reference for inclusion criteria, and the other two attended less than 85% of the training sessions.

### 2.3. Design of the Experiment

**Figure 1:**
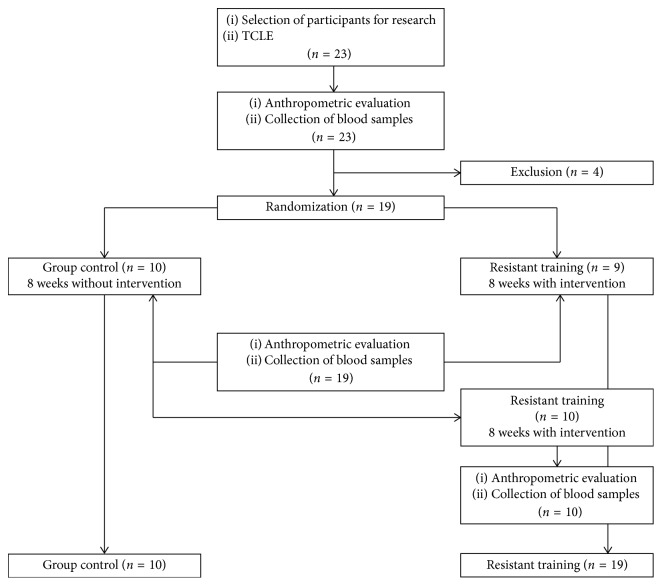
Study design flowchart.

A pretest/posttest with a control group design was adopted for the study. Both groups were pretested and posttested at eight weeks. The participants were randomly (http://www.randomization.com, Protocol no. 2538 08/2014, available on 08/18/2014) divided into the control group (Control) and experimental group (RT). The Control received no intervention, and the RT performed resistive training. It happened this way: first, the entire sample (*n*=19) was submitted to anthropometric assessment and blood collection; then, the entire sample was randomly assigned. One group (*n*=9) performed RT, while the other (*n*=10) received no intervention. After 24 hours, corresponding to eight weeks, all participants returned for a new anthropometric evaluation and blood collection. Then, the Control group started the intervention (*n*=10), performing RT for eight weeks as well. Afterwards, they were again submitted to anthropometric evaluation and blood collection. Then, the groups that performed RT were paired, forming the RT group (*n*=19) and the Control group (*n*=10) (Figure 1).

### 2.4. Resistive Training Program

The RT protocol was based on a critical analysis of the databases in [13, 18–20]. Thus, the training sessions were performed on fitness equipment for eight weeks. The type of training was the bi-set method, which consists of two exercises without time intervals for different muscle groups: lower limbs (LL) and upper limbs (UL).

For the protocol procedure, before and after all training sessions, participants rested for 5 minutes and then blood pressure was measured. The RT was supervised by one physiotherapist and two physical education professionals. The model of resistance training was by means of 8–12 maximal repetitions (MRs), aiming at muscular hypertrophy. In relation to the weekly frequency, the training protocol was performed three times per week, where eight exercises were performed in LL and UL: sitting leg press, sitting supine, knee extension, pulley (back), lying down knee flexion, low pulley elbow flexion, seated leg press, and pulley elbow extension. As for the increase in intensity (load (kg)), two criteria were adopted: (1) exercise intensity and load increase were obtained using the BORG scale; in other words, participants were instructed to mention any number from this scale, provided that the number actually corresponded to their perceived effort after each exercise series was performed. (2) After that, all participants performed their RT program within a maximum repetition (MR) zone of 8 to 12 repetitions; thus, whenever the participants exceeded the limits of this zone, a new increase of load occurred which would remain within the established zone, corresponding to 15% of the initial load. The time interval between the repetitions was one minute for each series, and the duration of the training protocol sessions lasted on average 50 minutes. In relation to the speed of execution of the exercise preestablished in four seconds for each movement, the repetition rate was performed with a concentric action of 2 seconds and an eccentric action of 2 seconds, controlled by visual and verbal commands to standardize angles of movement. The intervention of resistance training was very intense, and the participants were elderly and had not trained for at least six months, so in order to avoid possible side effects and possible withdrawal of the participants in the research, we chose to do a one-week adaptation training in the participants performed two sets of 15 submaximal repetitions with low loads, aiming at neuromuscular adaptation avoiding late-onset muscular pain.

### 2.5. Procedures and Data Collection

#### 2.5.1. Anthropometry and Body Composition

All participants were submitted to anthropometric assessment in pretest and posttest (eight weeks later). Body mass was measured using a digital scale (Welmy® W300, Brazil) with a maximum capacity of 300 kg. Height was measured using an anthropometric scale between 1.00 and 2.00 m. These measurements were used to calculate the body mass index (BMI). Also, waist and hip circumference was measured using the metric tape (Waist Fit®, Brazil) to calculate the waist-to-hip ratio. Prior to the measurements of body composition, participants were instructed to not eat 2-3 hours before, not drink alcohol, not perform any physical exercise for 24 hours, monitor fluid intake, and urinate 30 minutes prior to assessment. To begin the assessment, the participants were asked to lie down on the hammock, and then electrodes were put at predetermined points which were sanitized using 70% alcohol.

The emitter electrodes were placed at the dorsal surface of the right hand, near the metacarpophalangeal joint, and at the transverse arch of the upper surface of the right foot. The detector electrodes were placed between the distal prominences of the radius and ulna of the right wrist, and another one was placed between the medial malleolus and lateral malleolus of the right ankle [21]. This procedure was performed using a four-pole electrical bioimpedance (Maltron BF-906 Body Fat Analyzer®, England), whose technique is based on the fact that tissues with high water and electrolyte contents have high electrical conduction capacity and tissues with low water concentration show lower electrical conduction capacity; thus, this difference creates a good predictor for body parameters [5, 22]. All these procedures were performed at LAFIPEMA-UFMA at pretest (Pre) and posttest (Post), eight weeks after pretest.

#### 2.5.2. Blood Collection

Blood samples were collected by a trained pharmaceutical and in accordance with the biosafety standards recommended by NR32. The blood collection was performed at Pre (fasting) and Post (24 hours after the last day of RT) moments. At the moment Pre, all participants were instructed to go to the Laboratory of Physiology and Exercise Prescription (LAFIPEMA-UFMA), at 6 am, fasted for 8–12 hours. At the time Post, participants in the Control group made blood collections after a time period of 8 weeks. Already, the participants who performed RT made blood collections 24 hours after the last day of training. A total volume of 14 ml, approximately, was collected at vacuum and then distributed into a 4 ml EDTA tube (Vacuette) and two dry tubes containing 5 ml separating gel (Vacuette). The samples collected proceeded with the following usage: (a) the tube containing the EDTA anticoagulant was used to perform the complete blood count and the analysis of TNF-*α* gene expression and (b) the tubes containing the separating gel were used to perform the biochemical analysis of the high-sensitivity CRP and the ELISA immunoassays for cytokines IL-6 and TNF-*α*.

### 2.6. Analysis of Biochemical Parameters of High-Sensitivity CRP

This was analyzed to measure the CRP serum levels and feature the degree of risk for cardiovascular diseases. Therefore, biochemical analysis was performed by immunoturbidimetry.

### 2.7. Analysis of Cytokines IL-6 and TNF-*α*

#### 2.7.1. Serum Concentrations of IL-6 and TNF-*α*

The analysis of the blood concentrations of IL-6 and TNF-*α* was performed through ELISA tests at the Laboratory of Immunology and Microbiology of Respiratory Infections (LAMIR), CEUMA University. To process the immunoassay, serum aliquots and the IL-6 ELISA kits (SIGMA-ALDRICH 3050™, USA) and TNF-*α* ELISA kits (EIA-4641-DRG®, USA) were used, according to the manufacturer's instructions. This technique was performed using IL-6 and TNF-*α* monoclonal antibodies (MAbs), marked with enzymes, for chromogenic reactions. The quantification of IL-6 and TNF-*α* concentrations was performed by calorimetry, measuring the absorbance proportional to the concentration of each cytokine studied. A calibration curve was drawn, and the concentrations of both cytokines (IL-6 and TNF-*α*) were determined by interpolation from the curve.

All concentrations of IL-6 and TNF-*α* were determined by an enzyme immunoassay, using a microplate reader device with an 8-channel optical photometric system to measure the optical density of vertical light (Thermoplate® model TP Reader Basic, Brazil).

### 2.8. Analysis of TNF-*α* Gene Expression

#### 2.8.1. Total RNA Extraction and cDNA and TNF-*α* Preparation

The RNA isolation was performed from blood leukocytes collected in a vacuum tube containing the EDTA anticoagulant. A sample volume of 20 *μ*L was used for RNA extraction, according to the protocol using the GeneJET RNA Purification Kit (Thermo Scientific®, USA), in line with the manufacturer's recommendations. The total RNA was resuspended in 20 *μ*L of RNase-free water and quantified using a GeneQuant 100 spectrophotometer (GE Healthcare, Wisconsin, USA); after that, cDNA was synthesized from 200 *μ*g of total RNA, using the RevertAid First Strand cDNA Synthesis Kit (Thermo Scientific®, USA), according to the manufacturer's recommendations. In short, approximately 200 *μ*g of total RNA was reverse transcribed in a final volume of 20 *μ*L of reaction. The resulting cDNA was stored at −30°C until real-time PCR analysis.

#### 2.8.2. Real-Time Quantitative PCR Assay for TNF-*α*

Real-time quantitative PCR assays were performed for TNF-*α* gene analysis from blood leukocytes. A pair of primers presenting the following sequences were synthesized: TNF-*α*-5′-GTAGGACCCTGGAGGCTGAAC-3′ (sense) and 5′-CCCAAAAGAAATGGAGGCAAT-3′ (antisense), and GAPDH 5′-GGAAGGTGAAGGTCGGAGTCA-3′ (sense) and 5′-CTGGAAGATGGTGATGGGATTTC-3′ (antisense). Their construction was based on the gene sequences described by Schattner et al. [23]. GAPDH mRNA was used as the endogenous reference gene. Relative expression was calculated using the 2-ΔCt method. The real-time PCR was performed using the QuantStudio™ 6 Flex Real-Time PCR System (Thermo Fisher Scientific, USA). The reaction mix contained 2 *μ*L of the cDNA sample, 1.0 *μ*L of forward and reverse primers (200 nM), 10 *μ*L of Supermix, and 7.0 *μ*L of nuclease-free water. The oligonucleotides used in PCR were selected using the Primer Premier 6.1 Program (Premier Biosoft International®, USA). The amplicons were generated under the following thermocycling conditions: 1 cycle at 50°C for 2 min (UNG activation), 1 cycle at 95°C for 10 min (UNG inactivation), and 40 cycles at 95°C for 15 s and 60°C for 1 min.

### 2.9. Statistical Data Analysis

The statistical analysis was performed using the software GraphPad Prism 6.0. First, the Shapiro–Wilk normality test was performed, considering parametric tests (*p* > 0.05). The descriptive analysis of the variables, between the groups, was presented as mean and standard deviation, followed by Student's *T*-test for independent samples. To compare the anthropometric variables, body composition and lipid profile, at Pre and Post moments, the paired Student's *T*-test was performed. A one-way ANOVA test—paired for repeated measures and followed by Tukey's post hoc test—was performed to compare dependent samples of the load increase in the RT group. A two-way ANOVA test was performed to compare outcome variables between intra- and intergroups. The significance level was set at *p* < 0.05.

## 3. Results

### 3.1. Sample Characterization

Both groups presented similar variables, except for height (*p*=0.01) and body weight (*p*=0.04) (Table 1).

Comparing Pre and Post moments for RT and Control groups, there was a statistically significant difference in the RT group for the variables: decreased fat mass (*p*=0.02), body fat percentage (*p*=0.01), and WHR and increased lean mass (*p*=0.02), lean mass percentage (*p*=0.01), and body weight (Table 2).

Following the load evolution at the 1st, 4th, and 8th training weeks, there was a load increase in their respective exercises with a significant difference between the weeks (*p*=0.0001) (Table 3).

**Figure 2:**
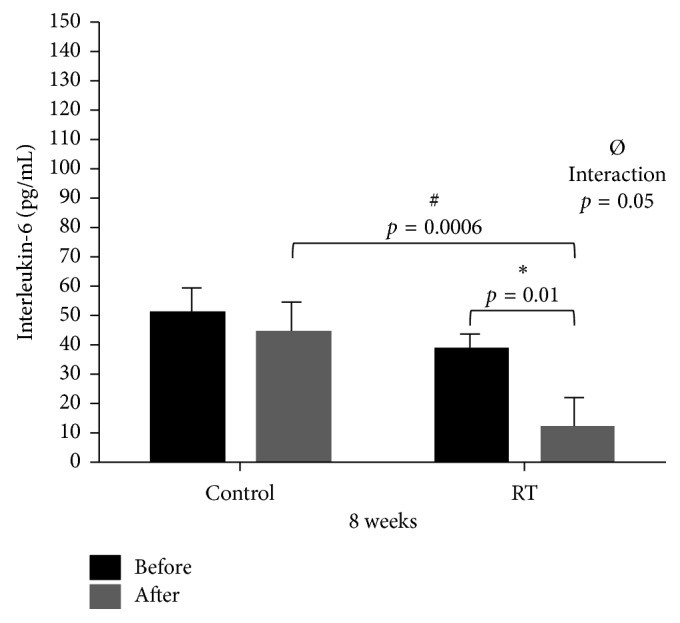
Dispersion measurements of interleukin-6 between Control (*n*=10) and RT (*n*=19) groups at Pre and Post moments. Two-way ANOVA test: ^*∗*^statistically significant difference (*p*=0.01) between Pre and Post moments in the RT group; ^#^statistically significant difference (*p*=0.0006) between Post moments in the RT vs Control groups; ^Ø^statistically significant interaction (*p*=0.05).

With respect to serum concentration of IL-6, a statistically significant interaction (*p*=0.05) between the groups (Control vs RT) and their respective moments (Pre vs Post) was noted, as well as a significant reduction (*p*=0.01) in the RT group between their respective moments (Pre vs Post) **(**Figure 2).

**Figure 3:**
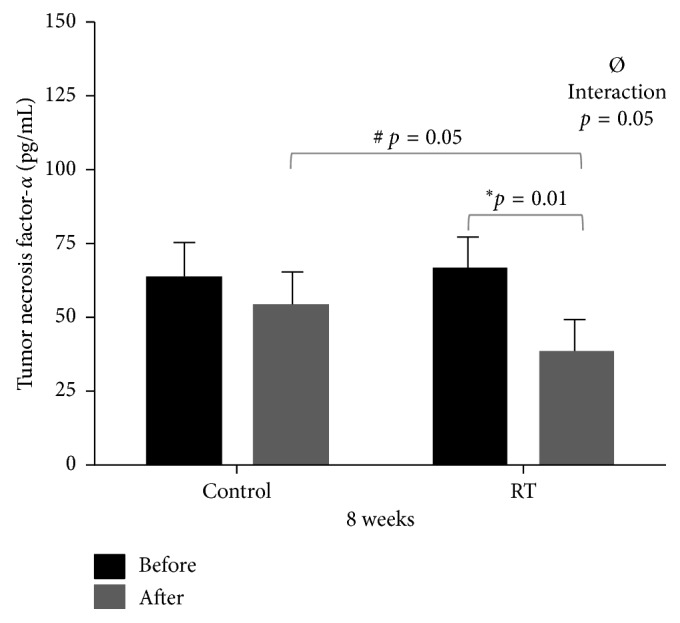
Dispersion measurements of tumor necrosis factor-*α* between Control (*n*=10) and RT (*n*=19) groups at Pre and Post moments. Two-way ANOVA test: ^*∗*^statistically significant difference (*p*=0.01) between Pre and Post moments in the RT group; ^#^statistically significant difference (*p*=0.05) between Post moments in the RT vs Control groups; ^Ø^statistically significant interaction (*p*=0.05).

With respect to serum concentration of TNF-*α*, there was a statistically significant interaction (*p*=0.05) between the groups (Control vs RT) and their respective moments (Pre vs Post), as well as a significant reduction (*p*=0.01) in the RT group between their respective moments (Pre vs Post) (Figure 3).

**Figure 4:**
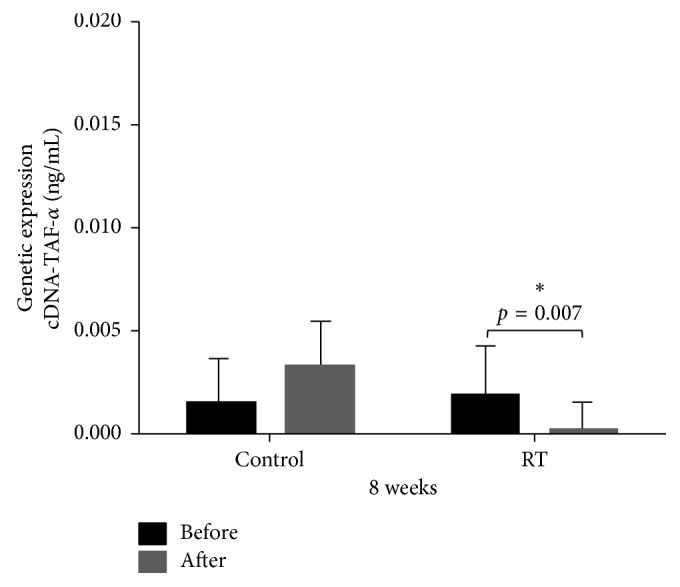
Measurements of tumor necrosis factor-*α* gene expression between Control (*n*=4) and RT (*n*=5) groups at Pre and Post moments. Two-way ANOVA test: ^*∗*^statistically significant difference (*p*=0.007) between Pre and Post moments in the RT group.

The molecular analysis of TNF-*α* gene expression between groups (Control vs RT), showed that although Pre values in the RT group were higher than the Pre values in the Control group, these values did not interfere in the statistically significant decrease observed in the RT group (*p*=0.007) (Figure 4).

**Figure 5:**
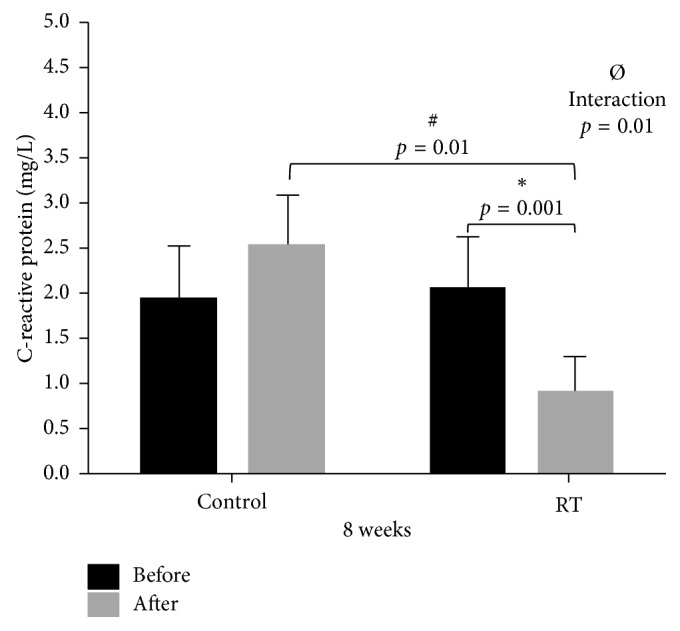
Dispersion measurements of C-reactive protein between Control (*n*=10) and RT (*n*=19) groups at Pre and Post moments. Two-way ANOVA test: ^*∗*^statistically significant difference (*p*=0.001) between Pre and Post moments in the RT group; ^#^statistically significant difference (*p*=0.01) between Post moments in the RT vs Control groups; ^Ø^statistically significant interaction (*p*=0.01).

With respect to serum concentration of CRP, there was a statistically significant interaction (*p*=0.01) between the groups (Control vs TR) and their respective moments (Pre vs Post), as well as a significant reduction (*p*=0.001) in the RT group between their respective moments (Pre vs Post) (Figure 5).

## 4. Discussion

This study aimed to evaluate the effects of eight weeks of RT on the immune response of IL-6 and TNF-*α*, the inflammatory marker of high-sensitivity CRP, and the TNF-*α* gene expression, as well as to analyze body composition in elderly women. Thereby, the initial hypothesis was confirmed, and there were observed a decrease in the serum concentrations of IL-6, TNF-*α*, and CRP after eight weeks of TR and a decrease in cellular activation, translated into the TNF-*α* gene expression. In addition, we observed a decrease in fat mass and an increase in lean mass and training load.

The objective of this study was to evaluate the behavior of the immune system of IL-6 and TNF-*α* and inflammatory marker of high-sensitivity PCR, as well as the gene expression of TNF-*α* in elderly women after eight weeks of intervention through the resistance training protocol. Thus, the initial hypothesis was confirmed, and a decrease in the serum concentrations of IL-6, TNF-*α*, and CRP after eight weeks of RT and a decrease in the cellular activation, translated into expression of the TNF-*α* gene, were observed. In addition, we observed a decrease in fat mass and an increase in lean mass, along with an increase in strength represented by the evolution of load along the resistance training.

In this study, eight weeks of RT significantly decreased the serum concentrations of IL-6, TNF-*α*, and CRP in the elderly women. TNF-*α* and IL-6 are cytokines produced by immune, endothelial, adipose, and smooth and skeletal muscle cells [24, 25]. Their main functions are to modulate innate and acquired immunity and regulate the response of the body during inflammatory processes [6]. These cytokines (IL-6 and TNF-*α*) have receptors on several cell types, including hepatocytes, and signal the synthesis of acute-phase proteins, such as CRP [5]. The TNF-*α* also stimulates the process of phagocytosis to repair and regenerate tissue [10, 24, 27]. For Abramson and Vaccarino [28], Costa and Vaisberg [29], Petersen and Pedersen [25], and Rossetti et al. [30], physical exercise is associated with muscle cell injury and, hence, the synthesis of anti-inflammatory cytokines (IL-6 and IL-10), from the bone marrow into the bloodstream, in balance with proinflammatory cytokines (TNF-*α* and IL-1Ra). The IL-6 has an important function in this muscle repair, since it increases soon after the beginning of the exercise and promotes anti-inflammatory actions and also inhibitory effects on the production and secretion of TNF-*α* (mainly), which cause a chronic adaptation to exercise [14].

Therefore, to analyze the chronic effects of physical exercise on inflammatory markers, it is essential to understand the acute effects of exercise on cytokine concentrations. Pereira et al. [31] investigated the effects of a single RT session on the serum concentrations of cytokines (TNF-*α*, IL-1a, IL-1b, IL-12, IL-6, and IL-10) in women with metabolic syndrome. No statistical difference was observed; however, there was a trend to decrease TNF-*α* concentration and to increase IL-1b and IL-6 concentrations 60 minutes after the training. This result indicates that a single RT session can modulate and reduce proinflammatory cytokines and shows that periodized and systematized training programs have a positive effect on cytokine concentrations. Rossetti et al. [30] explain that RT releases myokines (cytokines produced in active muscles), particularly IL-6, stimulating the production of anti-inflammatory cytokines (IL-6 and IL-10), which in turn inhibit the production of the proinflammatory cytokine (TNF-*α*). This process occurs in acute and consecutive training sessions and supports the systemic decrease in serum concentrations of total cytokines and, hence, in the acute inflammatory marker (CRP) [32, 33].

This was observed by Córdova et al. [14], who investigated the association between eight weeks of RT and serum concentrations of IL-6, TNF-*α*, and IFN-*γ* in elderly women. The study demonstrated that RT in elderly women is associated with low serum concentrations of proinflammatory cytokines, evidenced the impact of RT on these markers, and showed that sedentary lifestyle is a risk factor for diseases related to the aging process. Ho et al. [34] observed the effects of 12 weeks of training on inflammatory markers TNF-*α*, IL-6, and CRP in overweight and obese individuals and reinforced that idea because all studied variables decreased in the training group compared with the control.

Searching evidence about the molecular mechanisms behind these findings, it was verified that RT produces modulatory effects on TNF-*α* gene expression, influencing this proinflammatory mediator production. The results of the present study showed a significant decrease in TNF-*α* gene expression after 8 weeks of training, and this may be related to immunomodulation mediated by IL-6, since this cytokine acts as a natural TNF-*α* antagonist during long-term physical exercise and also promotes stimulation of anti-inflammatory cytokines production, like IL-1 antagonist receptor (IL-1Ra) and interleukin-10 (IL-10) [5, 6, 25].

In order to understand the control of TNF-*α* gene expression in terms of molecular mechanisms, Keller et al. [35] used an experimental model of induction of physical activity with *knockout* mice for the two main signaling pathways: TNF-*α* receptor 1 (p55) and TNF-*α* receptor 2 (p75). After training, TNF-*α* gene expression levels decreased in both *knockout* models, thus indicating that the activation of TNF-*α* receptors is one of the main mechanisms by which physical exercise regulates the TNF-*α* gene expression, although it is believed that other pathways may also be involved in this regulation.

As well, it is important to point out that the RT group presented significant gains in lean mass and loss of fat mass. These findings may also have contributed to the suppression of TNF-*α* gene expression, since, in obesity, adipose tissue produces a low-grade systemic inflammatory state, characterized by an increase in transcription and elevated levels of circulating TNF-*α* mRNA [25, 35].

Based on these data, adipose tissue contributes to the increase in TNF-*α* and CRP concentrations, which reverberates in their increased serum concentrations [25, 36]. Physical exercise induces a reduction in fat mass, by the mobilization of lipids and stimulation of lipolysis, which is regulated by lipase and activated by beta-oxidative stimulation. Thus, it increases the uptake and oxidation of fatty acids by the skeletal muscle and serves as an energetic substrate by the glucose fatty acid cycle mechanism, which directly reflects on the reduction of adipose tissue and, hence, on the reduction in serum concentrations of inflammatory markers (IL-6, TNF-*α*, and CRP). This is beneficial because it has a protective effect against cardiovascular diseases [25, 37]. The research conducted by Willis et al. [16] corroborates the findings of this study once it evaluated the effects of eight months of aerobic, resistance, and concurrent training on the body composition in overweight and obese adults and showed that aerobic and concurrent training reduced fat mass. Another similar study was conducted by Ho et al. [34], in which they investigated the effects of those three training modalities (aerobic, resistance, and concurrent training), for 12 weeks, in participants aged 40–66 years, and noted a decrease in fat mass and an increase in lean mass. Lee et al. [15] followed the effects on body composition, CRP, IL-6, and TNF-*α*, caused by two training modalities (aerobic and concurrent training), for eight weeks, in elderly women, and they observed a reduction in fat mass and an increase in lean mass, especially in aerobic training. Considering inflammatory markers, concurrent training decreased serum concentrations of CRP, IL-6, and TNF-*α*, compared to aerobic training. Based on the previously presented studies, it is possible to speculate that the decrease in fat mass and the increase in lean mass, verified in this study, were determinant for the decrease in serum concentrations of TNF-*α*, IL-6, and CRP, in the elderly women.

In this research, an increase in muscle strength was also observed, evidenced by the progressive increase in training load and in muscle volume, over the eight weeks of training. According to Prestes et al. [38], an adjustment of the body to training overload induces physiological and structural changes (neural and muscular factors, respectively), which results in an increase in muscle strength. The increase in muscle volume and strength may directly influence the decrease in serum concentrations of inflammatory markers (IL-6, TNF*α*, and CRP), as these variables have a strong relationship with each other [32]. For Rossetti et al. [30] and Petersen and Pedersen [25], this relationship is due to the fact that resistance training has an important neuroimmunoendocrine function; among them, myokines are cytokines produced in active muscles. The IL-6 has its wide action in other areas of the body, by means of important effects of the physical exercise in the adaptive process, since the muscular fibers seem to produce it through an independent way, stimulating in the circulation the appearance of other anti-inflammatory cytokines, as to IL-10, which in turn inhibits the production of the inflammatory cytokine TNF-*α*, via TNF-*α* receptor, TNF-R.

Mavros et al. [39] and Santiago et al. [40] studied the effects of 12 months of RT on the CRP in the elderly and associated its reduction with changes in body composition. They concluded that RT reduced serum concentrations of CRP and associated it with increased skeletal muscle mass (*p*=0.01). Thereby, they corroborate the findings of the present study. Moreover, Lera et al. [42] observed the importance of 16 weeks of RT on body composition, blood glucose, and CRP in menopausal, sedentary, and overweight elderly women. Their results indicated that RT increased muscle strength and muscle mass and prevented increased glucose and CRP.

The systematized and progressive RT program is developed through appropriate and specific training purposes. Among several protocols available, the bi-set method shows an efficient proposal for a well-structured and individualized RT program, which follows the RT prescription recommendations for the elderly. In view of this, it was decided to determine the training intensity by means of an RM range between 8 and 12 [13, 18–20, 43]. The results of this study suggest that chronic RT reduces serum concentrations of cytokines (IL-6 and TNF-*α*) and CRP and also induces molecular changes in TNF-*α* gene expression, in elderly women.

A possible limitation of this study may be related to the representativeness of the sample, since it is a limited group of women who are available to participate in institutional care research. Another limitation of the study was in relation to the elderly women who have passed th0065 menopause period, and it is known that the hormonal changes that occur in the postmenopausal period can influence the responses to the cytokines through body composition, mainly in the fat weight. In this way, we controlled this limitation with the decrease of the fat weight with the resistance training in relation to the basal values.

In summary, the results of the present study showed that eight weeks of RT caused molecular changes in TNF-*α* gene expression, in the elderly women, as well as a decrease in serum levels of IL-6, TNF-*α*, and CRP. This may be related to an immunomodulation and anti-inflammatory effect, since RT releases myokines (cytokines produced in active muscles), particularly IL-6, thus acting as a TNF-*α* antagonist during exercise. Therefore, the performance and adaptation to chronic RT controlled inflammatory conditions, which resulted in a balanced action between pro- and anti-inflammatory effectors in different tissues, especially in the striated skeletal muscle.

## Figures and Tables

**Table 1:** Characteristics of study participants.

Variable	Control (*n*=10)	RT (*n*=19)	*p* value
Age (years)	63.0 ± 1.0	63.0 ± 2.0	0.08
Height (m)	1.4 ± 0.0	1.5 ± 0.0	0.01^*∗*^
Body mass (kg)	58.5 ± 3.8	63.7 ± 2.4	0.04^*∗*^
Fat percentage (%)	39.1 ± 2.6	39.3 ± 1.4	0.60
Lean mass percentage (%)	60.8 ± 2.6	60.7 ± 1.4	0.60

Data are presented as mean ± standard deviation. Student's *T*-test for independent samples: ^*∗*^statistically significant difference (*p* < 0.05) between RT and Control groups.

**Table 2:** Anthropometric characteristics, body composition and lipid profile, in RT and Control groups at Pre and Post (8 weeks) moments.

Variables	Control (*n*=10)		*p* value	RT (*n*=19)		*p* value
	Pre	Post		Pre	Post	
Body mass (kg)	58.5 ± 3.8	58.1 ± 3.8	0.9	63.7 ± 2.4	65.0 ± 2.5	0.1
Fat mass (kg)	22.8 ± 2.9	22.2 ± 3.0	0.2	25.0 ± 1.7	23.5 ± 1.7^*∗*^	0.02
Lean mass (kg)	35.6 ± 1.1	35.7 ± 1.0	0.8	38.6 ± 3.6	41.4 ± 4.1^*∗*^	0.02
Fat percentage (%)	39.1 ± 2.6	38.2 ± 2.7	0.9	39.3 ± 1.4	36.2 ± 1.5^*∗*^	0.01
Lean mass percentage (%)	60.8 ± 2.6	61.7 ± 2.7	0.5	60.7 ± 1.4	63.7 ± 1.5^*∗*^	0.01
BMI (kg/m^2^)	26.8 ± 1.7	26.0 ± 1.7	0.1	27.7 ± 0.9	27.5 ± 1.0	0.7
WHR (cm)	0.8 ± 0.0	0.8 ± 0.0	0.3	0.8 ± 0.0	0.8 ± 0.0	0.1

Data are presented as mean ± standard deviation. Student's *T*-test: ^*∗*^statistically significant difference (*p* < 0.05) compared to Pre moment. BMI: body mass index; WHR: waist-to-hip ratio.

**Table 3:** Evolution of training load in first, fourth, and 8th weeks of RT.

Exercises	RT (*n*=19)			Effect size (Δ)	*p* value
	1st week	4th week	8th week		
Seated leg press (kg)	20.7 ± 3.8	33.6 ± 1.1^*∗*^	45.48 ± 1.4^*∗*^^†^	6.3	0.0001
Elbow flexion (low pulley) (kg)	9.0 ± 1.8	13.8 ± 0.3^*∗*^	17.83 ± 0.4^*∗*^^†^	4.8	0.0001
Knee extension (kg)	9.9 ± 0.5	18.4 ± 0.6^*∗*^	25.90 ± 0.8^*∗*^^†^	28.0	0.0001
Supine seated (kg)	7.1 ± 0.3	13.8 ± 0.4^*∗*^	17.96 ± 0.7^*∗*^^†^	29.2	0.0001
Lying knee flexion (kg)	7.9 ± 0.4	15.5 ± 2.0^*∗*^	17.09 ± 0.5^*∗*^	22.1	0.0001
Pulley (back) (kg)	13.2 ± 0.5	21.9 ± 0.4^*∗*^	27.03 ± 0.5^*∗*^^†^	26.4	0.0001
Plantar flexion (seated leg press) (kg)	20.0 ± 0.7	33.38 ± 0.9^*∗*^	43.56 ± 1.2^*∗*^^†^	30.9	0.0001
Elbow extension (pulley) (kg)	8.6 ± 0.6	16.42 ± 0.4^*∗*^	20.20 ± 0.4^*∗*^^†^	19.0	0.0001

Data are presented as mean ± standard deviation. One-way ANOVA test followed by Tukey's post hoc test: ^*∗*^statistically significant difference (*p* < 0.05) compared to the first week; †statistically significant difference (*p* < 0.05) compared to the fourth week. Δ: effect size measures on the evolution of training load among the analyzed weeks. RT: resistance training.

## Data Availability

All data used to support the findings of this study are included within the article.
